# Family History and ASCVD Risk Among Different Age Groups: Cohort Study in China and the United Kingdom

**DOI:** 10.1016/j.jacasi.2025.10.031

**Published:** 2026-02-20

**Authors:** Chenxi Yuan, Chong Shen, Jianxin Li, Keyong Huang, Xueli Yang, Jichun Chen, Xiaoqing Liu, Jie Cao, Shufeng Chen, Tao Zhou, Ling Yu, Yingxin Zhao, Xianping Wu, Liancheng Zhao, Ying Li, Dongsheng Hu, Jianfeng Huang, Xiangfeng Lu, Fangchao Liu, Dongfeng Gu

**Affiliations:** a Department of Epidemiology, Fuwai Hospital, National Center for Cardiovascular Diseases, Chinese Academy of Medical Sciences and Peking Union Medical College, Beijing, China; b Key Laboratory of Cardiovascular Epidemiology, Chinese Academy of Medical Sciences, Beijing, China; c Department of Epidemiology and Health Statistics, School of Public Health, Hangzhou Medical College, Hangzhou, China; d Department of Epidemiology, Center for Global Health, School of Public Health, Nanjing Medical University, Nanjing, China; e Research Units of Cohort Study on Cardiovascular Diseases and Cancers, Chinese Academy of Medical Sciences, Beijing, China; f Tianjin Key Laboratory of Environment, Nutrition and Public Health; Department of Occupational and Environmental Health, School of Public Health, Tianjin Medical University, Tianjin, China; g Division of Epidemiology, Guangdong Provincial People's Hospital and Cardiovascular Institute, Guangzhou, China; h Department of Cardiology, Shengli Clinical Medical College of Fujian Medical University, Fujian Provincial Hospital, Fuzhou University Affiliated Provincial Hospital, Fuzhou, China; i Cardio-Cerebrovascular Control and Research Center, Institute of Basic Medicine, Shandong Academy of Medical Sciences, Jinan, China; j Sichuan Center for Disease Control and Prevention, Chengdu, China; k Department of Epidemiology and Health Statistics, College of Public Health, Zhengzhou University, Zhengzhou, China; l Department of Epidemiology and Health Statistics, School of Public Health, Shenzhen University Health Science Center, Shenzhen, China; m Shenzhen Key Laboratory of Cardiovascular Health and Precision Medicine, Southern University of Science and Technology, Shenzhen, China; n School of Public Health and Emergency Management, Southern University of Science and Technology, Shenzhen, China; o School of Medicine, Southern University of Science and Technology, Shenzhen, China

**Keywords:** age, atherosclerotic cardiovascular disease, family history, prospective cohort

## Abstract

**Background:**

Evidence on the association between family history and atherosclerotic cardiovascular disease (ASCVD) across age groups remains limited.

**Objectives:**

This study aimed to evaluate the relations of family history of ASCVD (FHA) with incident ASCVD and its predictive value across age groups in China-PAR (Prediction for Atherosclerotic Cardiovascular Disease Risk in China) and UK Biobank.

**Methods:**

A total of 117,640 Chinese and 457,781 UK adults were included from 2 population-based cohorts, with family history assured by face-to-face interviews with standardized questionnaires.

**Results:**

During median follow-ups of 6.0 (Q1-Q3: 5.7-11.4) years (China-PAR) and 11.8 (Q1-Q3: 11.0-12.5) years (UK Biobank), 4,681 and 26,913 ASCVD cases occurred. Generally, FHA was consistently associated with higher ASCVD risk, but the association weakened with age (*P*_interaction_ < 0.001). The strongest effect was in adults <45 years, with HRs of 1.48 (95% CI: 1.11-1.96) in China-PAR and 1.47 (95% CI: 1.23-1.76) in UK Biobank, which was transformed to 6.75 and 5.33 years ASCVD-free years lost at the index of 20 years, and this gap decreased to 3.40 and 1.42 years at the index of 80 years, respectively. Notably, sibling history conferred greater risk than parental history (*P*_heterogeneity_ < 0.001).

**Conclusions:**

In both populations, FHA is a key indicator for identifying high ASCVD risk, especially in younger individuals, with a stronger impact driven by sibling history. These findings highlight the importance of tailoring recommendations for identifying high-risk individuals based on family history, with consideration of different age groups.

Atherosclerotic cardiovascular disease (ASCVD), including stroke and coronary heart disease (CHD), remains a leading cause of mortality and disease burden in both China and worldwide.^1^, ^2^, ^3^ Identifying high-risk populations has important public health and economic implications for improving the prevention of ASCVD.^4^^,^^5^ Family history information obtained from standardized questionnaires remains the most accessible way of measuring the inherited component of a disease in clinical practice and they represent the overall interaction between environmental and genetic factors, offering the opportunity for both population-wide health promotion and targeted intervention in high-risk groups.^6^, ^7^, ^8^, ^9^

Despite previous guidelines and studies having recognized the critical value of family history of ASCVD (FHA) in primary prevention of ASCVD,^4^^,^^10^^,^^11^ existing data on the association between family history and ASCVD risk have been controversial, especially among different age groups. For example, some studies have reported that the association between family history and the risk of cardiovascular disease was stronger among young subjects than among older subjects,^12^, ^13^, ^14^ but others report similar associations^15^ or opposite trends across age groups.^16^^,^^17^ Meanwhile, the adverse effects of family history on CHD and stroke were not uniform in different age groups.^18^ Given that family history is a complex heterogeneous trait, it is conceivable that the influence of family history on ASCVD may change as the dominant role of genetics and environment and behavior patterns in ASCVD shift with age.^6^^,^^19^ However, few studies have investigated the association between FHA and ASCVD across different age groups. This left a research gap in whether an age-specific family history screening should be differentially applied to younger and older populations in public health and clinical practice.

In addition, differences in the relationship between different types of family history of ASCVD (maternal, paternal, and sibling history) and incident ASCVD are also of concern. Several studies suggest that maternal history of cardiovascular disease confers a higher cardiovascular risk in their offspring than a paternal history of cardiovascular disease,^20^, ^21^, ^22^ whereas other studies have found insignificant differences^23^^,^^24^ or even contrary conclusions.^11^^,^^25^ Sibling history was more strongly correlated with incident ASCVD than parental history,^26^ although the difference was not always observed.^14^

Thus, using data from the China-PAR (Prediction for Atherosclerotic Cardiovascular Disease Risk in China) and UK Biobank, we systematically illustrate the relations of FHA with incident ASCVD, stroke, and CHD, and also estimate the application value of family history across different age groups and different types, so as to provide facilitate effective identification and prevention interventions for people at high risk of ASCVD in specific age groups, which will have a profound and beneficial impact on public health in both China and the United Kingdom.

## Methods

### Study Population

**Figure 1:**
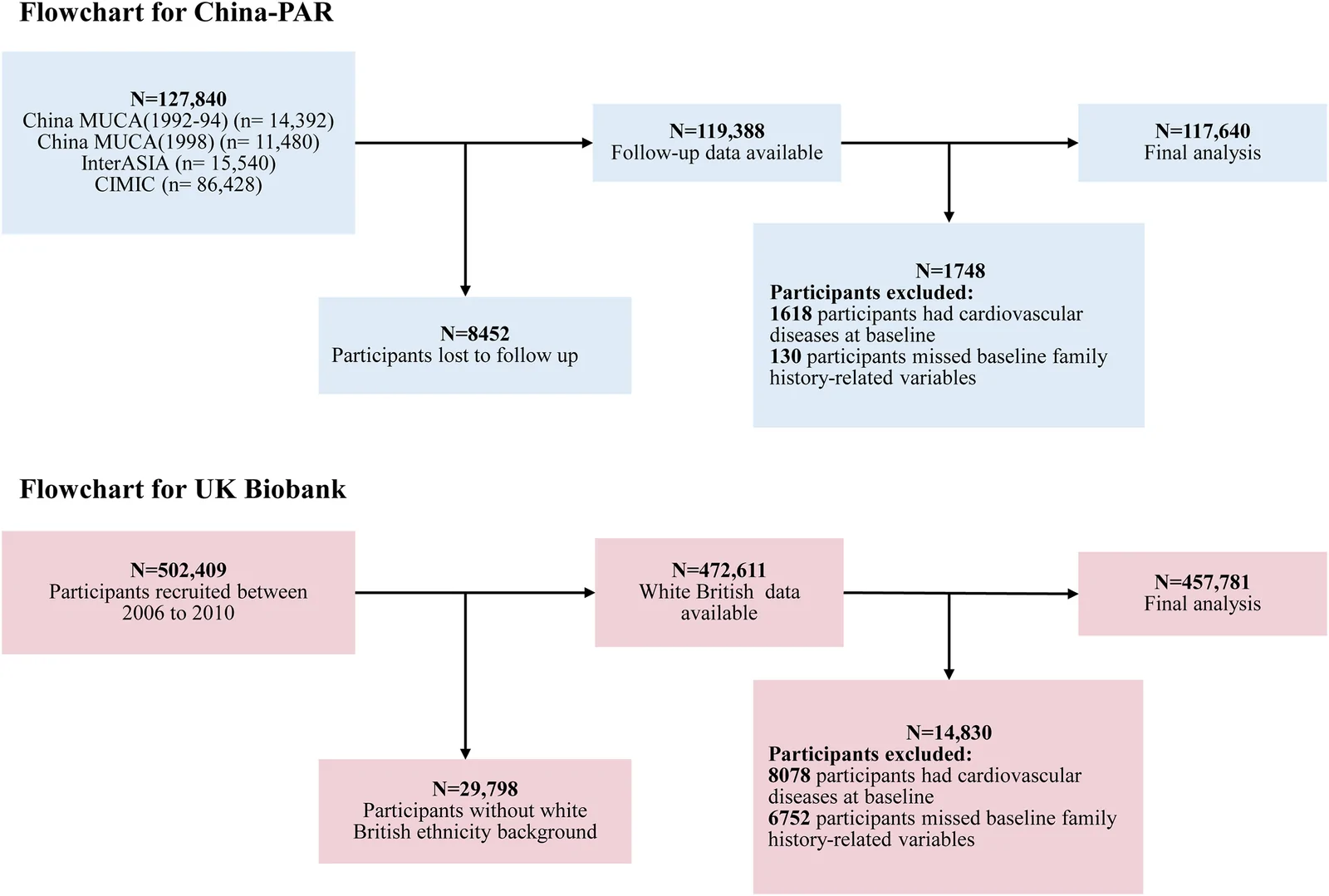
Flowchart of the Participants’ Inclusion for the Analysis Flowchart showing the selection of participants from the China-PAR (Prediction for Atherosclerotic Cardiovascular Disease Risk in China) and UK Biobank cohorts for analysis. Exclusions were applied for missing data, prior atherosclerotic cardiovascular disease events, or incomplete follow-up. Arrows indicate selection flow. China MUCA = China Multi-Center Collaborative Study of Cardiovascular Epidemiology; CIMIC = Community Intervention of Metabolic Syndrome in China and Chinese Family Health Study; InterASIA = International Collaborative Study of Cardiovascular Disease in Asia.

The China-PAR project is a largescale, prospective cohort with 127,840 enrolled participants aged 18 to 80 years from 15 Chinese provinces for the baseline examinations. A detailed description of these cohorts has been previously published.^27^^,^^28^ Briefly, participants were from 4 cohorts of the China-PAR project, including China MUCA (1992-1994) (China Multi-Center Collaborative Study of Cardiovascular Epidemiology 1992-1994), China MUCA (1998), InterASIA (International Collaborative Study of Cardiovascular Disease in Asia), and CIMIC (Community Intervention of Metabolic Syndrome in China and Chinese Family Health Study). According to a uniform protocol, the first follow-up survey for both InterASIA and the ChinaMUCA1998 was conducted from 2007 to 2008. The following follow-up survey was then conducted for all subcohorts in 2012 to 2015. The study timeline is presented in Supplemental Figure 1. Of the original study population, 119,388 participants (93.4%) completed the follow-up survey. After excluding participants with any ASCVD at baseline (n = 1,618) or missing information on family history-related variables (n = 130), 117,640 participants entered into the final analysis (Figure 1). This study was approved by the institutional review board at Fuwai Hospital in Beijing. Written informed consent was signed by each participant before data collection.

UK Biobank recruited over 500,000 middle-aged participants (age 37-73 years) from 22 assessment centers across England, Scotland, and Wales between 2006 and 2010. Details of the study design and data collection have been reported previously.^29^ Briefly, participants provided a wide range of health-related information through questionnaires on touch screen, physical examinations, and biological samples. Among the 502,409 participants, we excluded those who are not British ethnicity (n = 29,798), or had ASCVD at baseline (n = 8,078), or had missing information on family history–related variables (n = 6,752). Overall, 457,781 participants were included (Figure 1). The UK Biobank study was ethically approved by the North West Haydock Research Ethics Committee (reference number: 11/NW/0382) and carried out in accordance with the Declaration of Helsinki. All participants provided written informed consent to participate in the study.

### Assessment of Family History Status

In China-PAR and UK Biobank, each subject was asked by the interviewer during the baseline survey whether the first-degree relatives (father, mother, or siblings) had been affected by stroke or CHD. We defined a participant as having FHA if he or she reported that 1 parent or sibling had a stroke and/or CHD. Positive parental history was defined as reporting a positive history for either or both parents. In this study, first-degree relatives included fathers, mothers, and siblings.

### Assessment of Covariates

We selected potential confounders according to the literature and expert knowledge, and identified the minimal sufficient adjustment set, including age, sex, current smoker, alcohol drinker, ideal physical activity, waist circumference, fasting glucose level (China-PAR) or hemoglobin A_1c_ (UK Biobank), total cholesterol, high-density lipoprotein cholesterol, and systolic blood pressure, using the directed acyclic graph^30^ (Supplemental Figure 2). In the China-PAR, a team of trained personnel utilized standardized questionnaires to collect demographic, lifestyle, and medical history data from each participant at baseline. Smokers were those who consumed at least 400 cigarettes or 500 g of tobacco in a lifetime or smoked at least 1 cigarette a day for more than 1 year, and those who were smoking at the time of survey were defined as current smokers. Alcohol drinkers was defined as drinking alcohol at least once a week in the past 1 year. Physical activity was classified into ideal or non-ideal status, depending on whether the participants engage in at least 150 min/wk of moderate physical activity or 75 min/wk of vigorous physical activity or an equivalent combination of both. Waist circumference was measured at a point located 1 cm above the navel at minimal respiration. After fasting for at least 10 hours, blood samples were taken from participants, and serum glucose and lipids levels were measured under uniform quality control standards. Diabetes was defined as a fasting glucose level of ≥126 mg/dL or using antidiabetic medication (such as insulin or oral hypoglycemic agents). Each participant's blood pressure was measured 3 times after a 5-minute rest, and the average was taken as the final analysis. In the current analysis, hypertension was defined as systolic blood pressure ≥140 mm Hg or diastolic blood pressure ≥90 mm Hg, or use of antihypertensive medication within the past 2 weeks. In UK Biobank, covariates were obtained through a touch-screen questionnaire and anthropometric assessment completed by participants at the time of recruitment. Smoking was defined in the questionnaire as smoking at least 100 cigarettes in life, and those who were smoking at the time of survey were defined as current smokers. Status, frequency, and volume of current alcohol consumption were self-reported, and alcohol consumption was defined as drinking at least once a week, including on special occasions. Physical activity was measured by duration of moderate and vigorous activity per week and was classified into ideal or non-ideal physical activity in line with China-PAR. Diabetes was defined as hemoglobin A_1c_ ≥6.5%, the use of antidiabetic medication, or self-reported doctor-diagnosed diabetes. Hypertension was defined as systolic blood pressure ≥140 mm Hg, diastolic blood pressure ≥90 mm Hg, use of antihypertensive medication, or self-reported doctor-diagnosed hypertension.

### Outcome Ascertainment and Definition

ASCVD events in the present study included nonfatal acute myocardial infarction, death resulting from CHD, or fatal or nonfatal stroke. ASCVD incidence was defined as the first ever ASCVD event. In the China-PAR, information regarding incidence during follow-up surveys was collected through interviewing study participants or their proxies and checking hospital records and/or death certificates to validate the diagnosis using International Classification of Diseases-10th Revision (ICD-10) codes. CHD, nonfatal acute myocardial infarction, and stroke were classified under ICD-10 codes I20-I25, I21, as well as I60, I61, I63, and I64, respectively. To ascertain disease status, a committee of experts at Fuwai Hospital was established to review all medical records or death certificates. Each endpoint was evaluated independently by 2 committee members and then discussed by other members in case of discrepancies. In UK Biobank, ASCVD diagnoses, including incident CHD (ICD-10 codes I20-I25), nonfatal acute myocardial infarction (ICD-10 code I21), and stroke diagnoses (ICD-10 codes I60, I61, I63, and I64), were obtained through linked hospital admissions data including Hospital Episode Statistics-Admitted Patient Care (England), Scottish Morbidity Records-General/Acute Inpatient and Day Case Admissions (Scotland), and Patient Episode Database for Wales, as well as death register data.

### Statistical Analysis

Baseline characteristics were described across different status of FHA. Continuous data were presented as mean ± SD, and categorical variables as number (frequency). We calculated person-years of follow-up from the date of initial examination to the occurrence of ASCVD onset, death, or the last follow-up (December 31, 2015, for China-PAR and December 30, 2020, for UK Biobank), whichever occurred first.

Cohort-stratified Cox proportional hazard regression model (for China-PAR) and Cox proportional hazard regression model (for UK Biobank) were used to estimate the HRs and 95% CIs of ASCVD, stroke, and CHD incident events according to the history of ASCVD in first-degree relatives (yes or no), and the number of first-degree relatives with ASCVD (no, 1, ≥2). The proportional hazards assumption for all Cox models was assessed using Schoenfeld residuals, and no significant violations were observed for any of the main exposures or covariates (*P* > 0.05). *P* values for trend were obtained from linear trend tests across these ordered categories. Besides, the HRs were evaluated according to types of family history (including father, mother, parents, and sibling history). Based on the directed acyclic graph (Supplemental Figure 2), we considered 2 models: model 1 included age and sex; model 2 was further adjusted for current smoker, alcohol drinker, ideal physical activity, waist circumference, fasting glucose level (China-PAR) or hemoglobin A_1c_ (UK Biobank), total cholesterol, high-density lipoprotein cholesterol, and systolic blood pressure. Stratified analyses were conducted to assess the effects of FHA across different age groups (<45 years, 45-60 years, ≥60 years). The multiplicative interaction analyses were performed by including the product terms of family history and age group into the Cox model. Moreover, the years of ASCVD-, stroke-, and CHD-free years lost related to the history of ASCVD in first-degree relatives compared with those without were estimated by calculating the differences of areas under the survival curves fitted by the fully adjusted Cox proportional model with age as the timescale. In order to estimate the 95% CI, bootstrap simulations were applied, which generated new bootstrapped samples in equal sample size from the original data with 500 replications. The 2.5th and 97.5th percentiles of these distributions were the 95% CIs.

Finally, we conducted several sensitivity analyses to assess the robustness of our findings by excluding events that occurred within the first year of follow-up to reduce potential reverse causation. In addition, Fine-Gray models were employed to account for the competing risk of death.^31^ We additionally adjusted for socioeconomic status (SES), which is a measure of an individual’s or family’s social or economic position or rank in a social group.^32^^,^^33^ Self-reported education level, employment status, and household income level were applied to assess SES according to previous studies.^34^^,^^35^ Participants were categorized as low SES, medium SES, and high SES, respectively. Details are reported in the Supplemental Appendix.

All statistical tests were 2 sided, with a *P* value of 0.05 considered to be significant. We used SAS (version 9.4, SAS Institute) and R software (version 3.3.0, R Foundation for Statistical Computing) for the analyses.

## Results

### Baseline Characteristics

Table 1 shows baseline characteristics of participants from the China-PAR and UK Biobank. Among 117,640 participants from China-PAR (mean age 50.9 years), 41.1% were men (48,327/117,640), and 19,199 had FHA. Among 457,781 participants from UK Biobank (mean age 56.7 years), 45.0% were men (206,002/457,781), and 215,006 had FHA. Compared with participants without FHA, adults with FHA were more likely to be older, hypertensive, and diabetic, as well as to have unhealthy levels of diet, waist circumference, total cholesterol, and high-density lipoprotein cholesterol in China-PAR, and those in UK Biobank were more likely to have a higher total cholesterol levels and lower SES, and they tended to have non-ideal physical activity, hypertension, or diabetes.

**Table 1:** Baseline Characteristics of Participants From the China-PAR and UK Biobank

	China-PAR (N = 117,640)			UK Biobank (N = 457,781)		
	Total Population (N = 117,640)	Family History of ASCVD		Total Population (N = 457,781)	Family History of ASCVD	
		No (n = 98,441)	Yes (n = 19,199)		No (n = 242,775)	Yes (n = 215,006)
Age, y	50.9 ± 11.8	50.6 ± 11.0	52.3 ± 10.6	56.7 ± 8.0	55.6 ± 8.4	57.9 ± 7.4
Age groups, y						
<45	40,976 (34.8)	35,381 (35.9)	5,595 (29.1)	44,829 (9.8)	32,115 (13.2)	12,714 (5.9)
45-60	50,245 (42.7)	41,266 (41.9)	8,979 (46.8)	211,718 (46.2)	114,665 (47.2)	97,053 (45.1)
≥60	26,419 (22.5)	21,794 (22.1)	4,625 (24.1)	201,234 (44.0)	95,995 (39.5)	105,239 (48.9)
Male	48,327 (41.1)	40,427 (41.1)	7,900 (41.2)	206,002 (45.0)	116,030 (47.8)	89,972 (41.9)
Healthy sleep^a^	65,396 (67.4)	55,522 (68.5)	9,874 (61.9)	311,589 (68.1)	167,831 (69.1)	143,758 (66.9)
Healthy diet^b^	28,311 (27.4)	23,971 (27.7)	4,340 (25.9)	149,768 (32.7)	77,461 (31.9)	72,307 (33.6)
Ideal physical activity^c^	68,374 (66.7)	57,086 (66.4)	11,288 (68.1)	186,401 (40.7)	99,708 (41.1)	86,693 (40.3)
Current smoker	28,066 (24.0)	23,467 (23.9)	4,599 (24.0)	47,208 (10.3)	26,458 (10.9)	20,750 (9.7)
Alcohol drinker	23,039 (19.6)	19,581 (19.9)	3,458 (18.0)	350,215 (76.5)	188,586 (77.7)	161,629 (75.2)
Waist circumference, cm	80.1 ± 10.3	79.7 ± 10.2	82.1 ± 10.1	90.1 ± 13.5	89.8 ± 13.4	90.5 ± 13.6
Hypertension	37,511 (31.9)	29,785 (30.3)	7,726 (40.3)	239,807 (52.4)	116,370 (47.9)	123,437 (57.4)
Diabetes mellitus	6,107 (5.5)	4,740 (5.1)	1,367 (7.4)	27,391 (6.0)	12,688 (5.2)	14,703 (6.8)
Total cholesterol, mg/dL	175.3 ± 35.6	174.7 ± 35.6	178.2 ± 35.4	221.5 ± 43.7	221.5 ± 43.3	221.9 ± 44.5
High-density lipoprotein cholesterol, mg/dL	51.3 ± 13.0	51.6 ± 13.0	49.7 ± 12.6	56.4 ± 14.7	56.4 ± 14.7	56.1 ± 14.7
Socioeconomic status^d^						
Low	52,090 (45.4)	43,672 (45.5)	8,418 (44.7)	121,330 (31.1)	57,566 (27.7)	63,764 (35.1)
Medium	35,222 (30.7)	28,927 (30.2)	6,295 (33.4)	151,809 (39.0)	82,364 (39.7)	69,445 (38.2)
High	27,445 (23.9)	23,324 (24.3)	4,121 (21.9)	116,326 (29.9)	67,746 (32.6)	48,580 (26.7)

Values are mean ± SD or n (%).

ASCVD = atherosclerotic cardiovascular disease; China-PAR = Prediction for Atherosclerotic Cardiovascular Disease Risk in China.

a Healthy sleep denoted an ideal sleep duration at least 7 hours and <9 hours per day.

b In China-PAR (Prediction for Atherosclerotic Cardiovascular Disease Risk in China), healthy diet denoted reaching ideal intakes of 3 or more kinds of food (fish, fruits and vegetables, soybean, red meat, and tea) for cardiovascular health. In UK Biobank, healthy diet denoted ideal intake of 5 or more dietary components for cardiovascular health.

c Ideal physical activity was defined as meeting the recommended moderate and vigorous physical activity (at least 150 minutes (min/wk) of moderate physical activity or 75 minutes of vigorous physical activity or an equivalent combination of moderate- and vigorous-intensity activity).

d In China-PAR, socioeconomic status was assessed by education level, occupation prestige, and household monthly per capita income. In UK Biobank, socioeconomic status was assessed by education level, employment status, and average total household income.

### Association of Family History with ASCVD Risk

In China-PAR, 4,681 incident ASCVD cases were recorded during a median follow-up of 6.0 years, and 26,913 incident ASCVD cases were recorded over a median follow-up of 11.8 years in UK Biobank. The incidence rate was 442 cases per 100,000 person-years (95% CI: 419-476; 4,681 events/106.0 million person-years) in Chinese participants, which was lower than that of 514 cases per 100,000 person-years (95% CI: 509-522 cases; 26,913 events/523.6 million person-years) in British participants. Generally, participants with FHA were significantly associated with higher risk of ASCVD, compared with those without FHA; HR was 1.41 (95% CI: 1.27-1.56) and 1.34 (95% CI: 1.30-1.38) in the Chinese and British, respectively (Table 2). Furthermore, association between FHA and risk of ASCVD strengthened with the increasing number of first-degree relatives with ASCVD. After multivariate adjustment in model 2, individuals with 1 and ≥2 first-degree relatives with ASCVD had HRs of 1.38 (95% CI: 1.23-1.54) and 1.59 (95% CI: 1.25-2.03), respectively, in China-PAR, as well as 1.26 (95% CI: 1.22-1.30) and 1.53 (95% CI: 1.47-1.59), respectively, in UK Biobank (*P* < 0.0001 for trend), respectively, compared with those without FHA. In Table 2, the HRs for stroke were increased with the number of first-degree relatives with ASCVD, which also was the case for CHD.

**Table 2:** Association Between Family History Status and Incident Risk of ASCVD, Stroke, and CHD

Group	Family History Status	China-PAR (N = 117,640)			UK Biobank (N = 457,781)		
		Events/Total	Model 1	Model 2	Events/Total	Model 1	Model 2
ASCVD							
	No	3,464/98,441	1.00 (ref)	1.00 (ref)	11,752/242,775	1.00 (ref)	1.00 (ref)
	Yes	1,217/19,199	1.50 (1.37-1.63)	1.41 (1.27-1.56)	15,161/215,006	1.37 (1.34-1.40)	1.34 (1.30-1.38)
	1 member	1,026/16,620	1.46 (1.33-1.59)	1.38 (1.23-1.54)	10,174/151,510	1.29 (1.25-1.32)	1.26 (1.22-1.30)
	≥2 members	191/2,579	1.77 (1.45-2.16)	1.59 (1.25-2.03)	4,987/63,496	1.59 (1.54-1.64)	1.53 (1.47-1.59)
	*P* for trend	—	<0.0001	<0.0001	—	<0.0001	<0.0001
Stroke							
	No	2,638/98,441	1.00 (ref)	1.00 (ref)	4,421/242,775	1.00 (ref)	1.00 (ref)
	Yes	1,023/19,199	1.52 (1.38-1.67)	1.44 (1.28-1.61)	5,269/215,006	1.20 (1.15-1.25)	1.18 (1.12-1.23)
	1 member	860/16,620	1.47 (1.33-1.63)	1.40 (1.24-1.59)	3,625/151,510	1.15 (1.10-1.21)	1.14 (1.08-1.20)
	≥2 members	163/2,579	1.82 (1.46-2.28)	1.69 (1.29-2.21)	1,644/63,496	1.30 (1.23-1.38)	1.28 (1.20-1.37)
	*P* for trend	—	<0.0001	<0.0001	—	<0.0001	<0.0001
CHD							
	No	885/98,441	1.00 (ref)	1.00 (ref)	8,279/242,775	1.00 (ref)	1.00 (ref)
	Yes	211/19,199	1.44 (1.24-1.66)	1.29 (1.08-1.54)	11,643/215,006	1.42 (1.38-1.45)	1.36 (1.33-1.40)
	1 member	182/16,620	1.35 (1.15-1.58)	1.21 (1.00-1.47)	7,685/151,510	1.32 (1.29-1.35)	1.28 (1.24-1.31)
	≥2 members	29/2,579	2.01 (1.47-2.74)	1.80 (1.25-2.59)	3,958/63,496	1.67 (1.62-1.72)	1.59 (1.53-1.64)
	*P* for trend	—	<0.0001	0.0007	—	<0.0001	<0.0001

Stratified Cox proportional hazard model in China-PAR and Cox proportional hazard model in the UK Biobank. Model 1: including age and sex. Model 2: adjusted for age, sex, current smoker, alcohol drinker, ideal physical activity, waist circumference, fasting glucose level (China-PAR) or hemoglobin A_1c_ (UK Biobank), total cholesterol, high-density lipoprotein cholesterol, and systolic blood pressure. *P* for trend: linear trend test *P* value for HRs’ across number of first-degree relatives (no, 1 member, and ≥2 members) having family history within each model.

CHD = coronary heart disease; other abbreviations as in Table 1.

**Figure 2:**
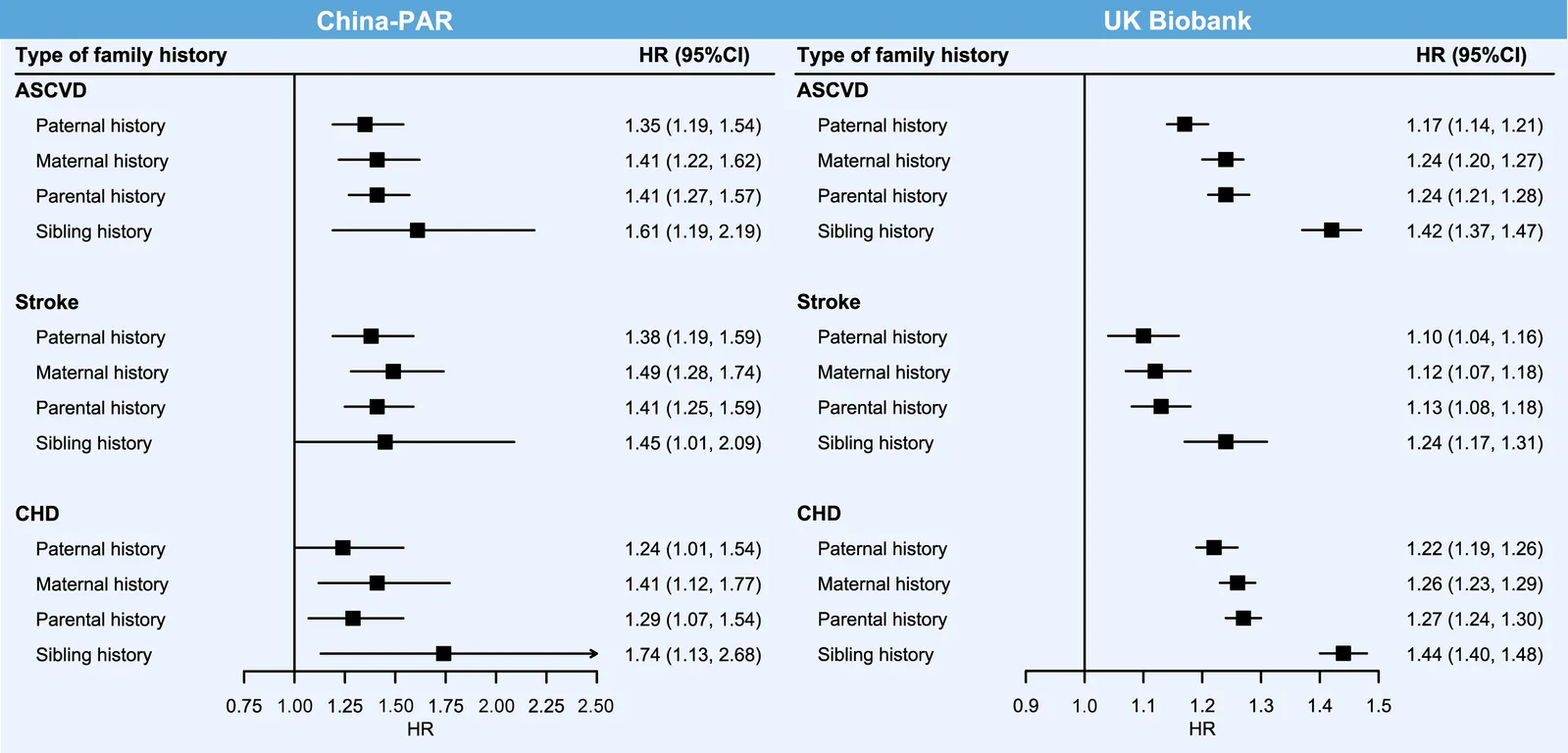
Association of Incident ASCVD, Stroke, and CHD With Family History Cox proportional hazard models were stratified by cohort (just in China-PAR) and adjusted for age, sex, current smoker, alcohol drinker, ideal physical activity, waist circumference, fasting glucose level (China-PAR) or hemoglobin A_1c_ (UK Biobank), total cholesterol, high-density lipoprotein cholesterol, and systolic blood pressure. ASCVD = atherosclerotic cardiovascular disease; CHD = coronary heart disease; other abbreviations as in Figure 1.

In addition, we further assessed the impact of different types of family history on the risk of developing ASCVD (Figure 2). After the multivariable adjustment, the HR was 1.61 (95% CI: 1.19-2.19) for sibling history and 1.41 (95% CI: 1.27-1.57) for parental history (*P*_heterogeneity_ < 0.001) in China, as well as 1.42 (95% CI: 1.37-1.47) and 1.24 (95% CI: 1.21-1.28), respectively, in the United Kingdom (*P*_heterogeneity_ < 0.001). Moreover, the HRs and 95% CIs of incident ASCVD relevant to paternal history and maternal history remained comparable (*P*_heterogeneity_ = 0.785 in China, *P*_heterogeneity_ = 0.089 in the United Kingdom). Similar patterns were also shown for both stroke and CHD in terms of the difference between sibling and parental history, and in terms of the consistency between paternal and maternal history. Of note, the ASCVD risk associated with family history, regardless of the type, was slightly higher in the Chinese population than that in the British.

### The Effect of Family History on Ascvd in Different Age Groups

**Figure 3:**
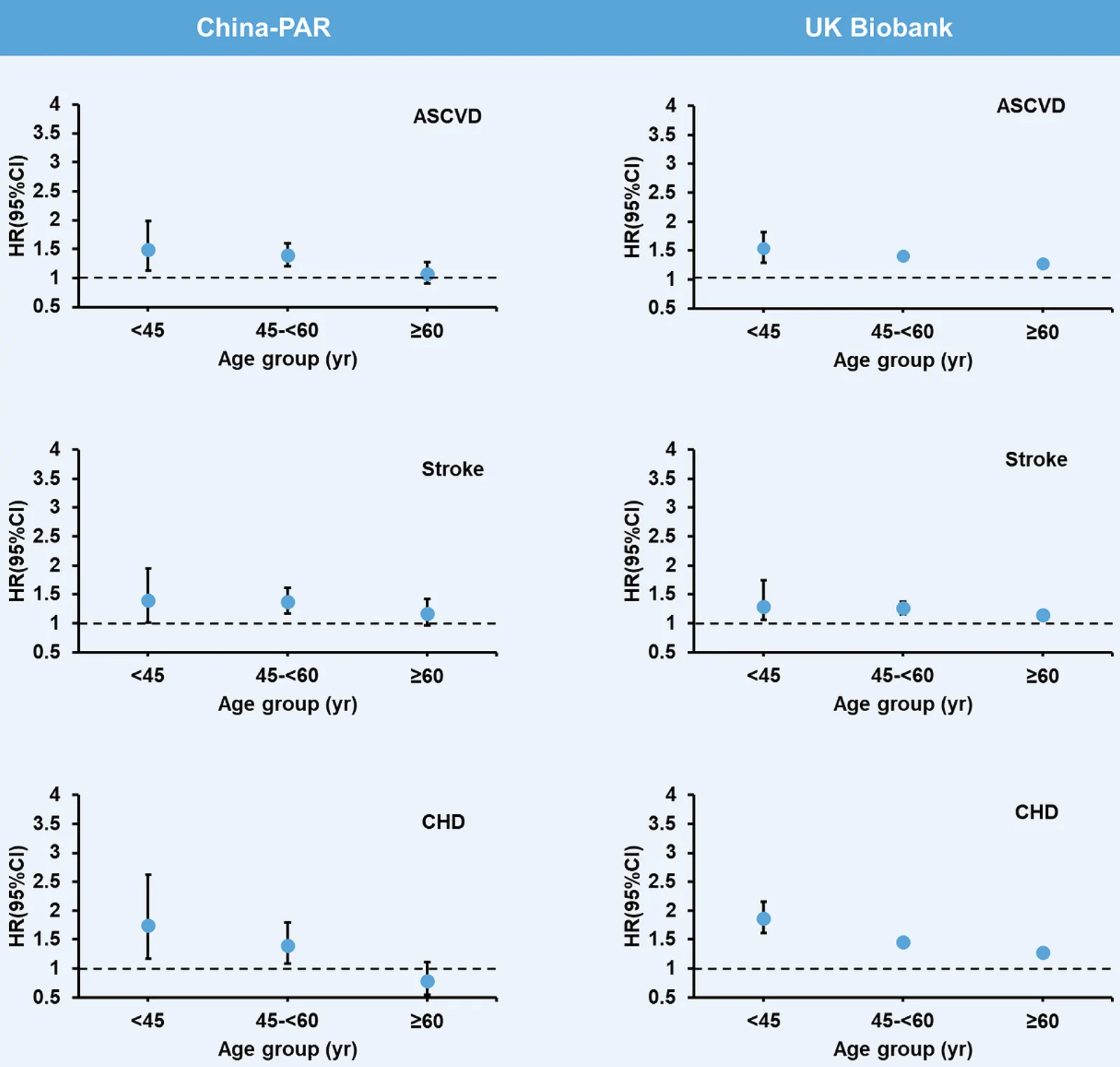
Age-Specific Risk of ASCVD, Stroke, and CHD by Family History HRs were stratified by cohort (just in China-PAR) and adjusted for sex, current smoker, alcohol drinker, ideal physical activity, waist circumference, fasting glucose level (China-PAR) or hemoglobin A_1c_ (UK Biobank), total cholesterol, high-density lipoprotein cholesterol, and systolic blood pressure. yr = year; other abbreviations as in Figure 2.

Stratified analyses according to age groups (classified as <45, 45-60, and ≥60 years) were further performed. We found that the effect size of incident ASCVD related to family history decreased with age (Figure 3). The strongest association was observed in the young (age <45 years), with HR of 1.48 (95% CI: 1.11-1.96) in China-PAR and 1.47 (95% CI: 1.23-1.76) in UK Biobank, whereas the smallest HR values were seen in the elderly (age ≥60 years), and among Chinese older adults over age 60 years, family history even disappeared as an independent risk factor for ASCVD (Supplemental Table 1). This trend was also obvious in both family history of stroke and CHD in Figure 3. Particularly, we observed an antagonistic effect of family history and age groups on the risk of ASCVD, both in Chinese and British (*P*_interaction_ < 0.001).

**Figure 4:**
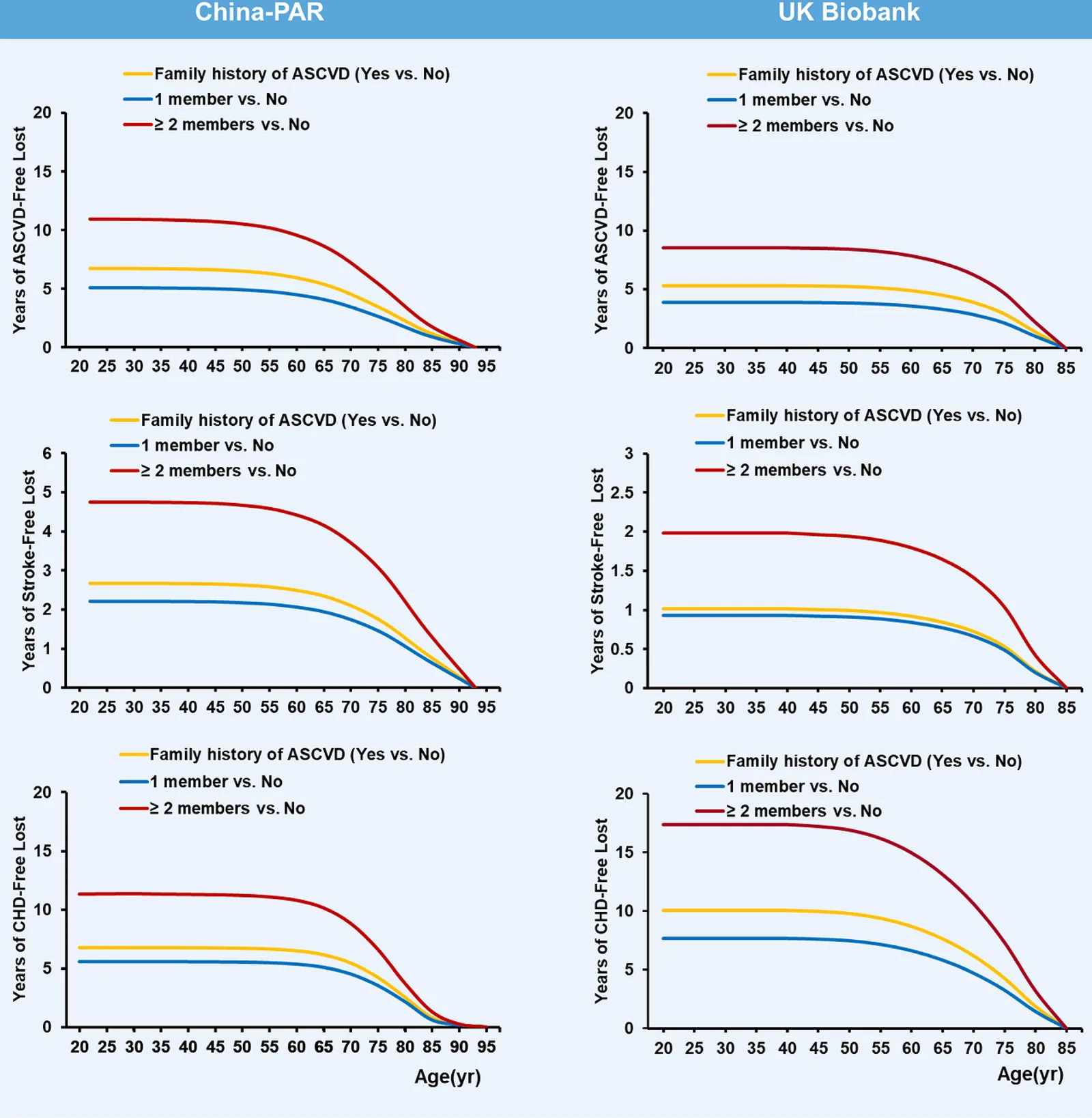
Cardiovascular Disease-Free Years Lost by Family History Cardiovascular disease–free years lost were evaluated in participants with a history of cardiovascular events in first-degree relatives compared with those without a family history at different ages. Models were stratified by cohort (just in China-PAR) and adjusted for age, sex, current smoker, alcohol drinker, ideal physical activity, waist circumference, fasting glucose level (China-PAR) or hemoglobin A_1c_ (UK Biobank), total cholesterol, high-density lipoprotein cholesterol, and systolic blood pressure. Abbreviations as in Figure 2.

To make the results more communicable, we further estimated the loss of ASCVD-, stroke-, and CHD-free years in people with FHA (Figure 4). In both China and the United Kingdom, participants with FHA were estimated to have fewer ASCVD-, stroke-, and CHD-free years compared with those without a family history, but this gap decreased with increasing age, which further suggested that the effect of family history on ASCVD decreases with age. As shown in Figure 4, those with FHA developed ASCVD, stroke, or CHD 6.75 (95% CI: 5.34-8.04) years, 2.69 (95% CI: 1.48-3.85) years, and 6.79 (95% CI: 5.41-7.97) years earlier than those without family history at the index age of 20 years, respectively, but this advance was reduced to 3.40 (95% CI: 1.89-4.92) years, 1.73 (95% CI: 0.38-3.13) years, and 2.55 (95% CI: 1.83-3.27) years at the index age of 80 years in China-PAR. Similarly, in UK Biobank, the difference in ASCVD-free years between people without family history and those with FHA or family history of stroke or CHD was 5.33 (95% CI: 4.42-6.23) years, 1.04 (95% CI: 0.83-1.25) years, or 10.06 (95% CI: 9.78-10.34) years, respectively, at the index age of 20 years, but the difference was reduced to 1.42 (95% CI: 1.01-1.83) years, 0.23 (95% CI: 0.18-0.28) years, or 1.88 (95% CI: 1.63-2.13) years, respectively, at the index age of 80 years.

### Sensitivity Analyses

Sensitivity analyses were performed to examine the association of FHA with ASCVD, stroke, and CHD risk in the general population and in 3 age groups (<45, 45-60, and ≥60 years), respectively. The results remain consistent after excluding participants who developed ASCVD within the first year of follow-up. In the competing risk analyses, the results from the Fine-Gray model were similar to the main results. Additionally, the associations persisted after further adjustment for SES, supporting the robustness of our main findings (Supplemental Table 2).

## Discussion

In these 2 large cohorts from China and the United Kingdom, individuals with a family history had increased risk of developing ASCVD, and the effect size was larger for those with an increasing number of first-degree relatives affected by ASCVD. Notably, the association was more pronounced among younger adults. Furthermore, by comparing the results of different types of family history, the effect of sibling history was more significant than that of parental history (Central Illustration). These findings provide robust evidence for the impact of family history on risk of ASCVD among different age groups and have important implications for age-specific primary prevention of ASCVD and clinical decision-making.

**Central Illustration:**
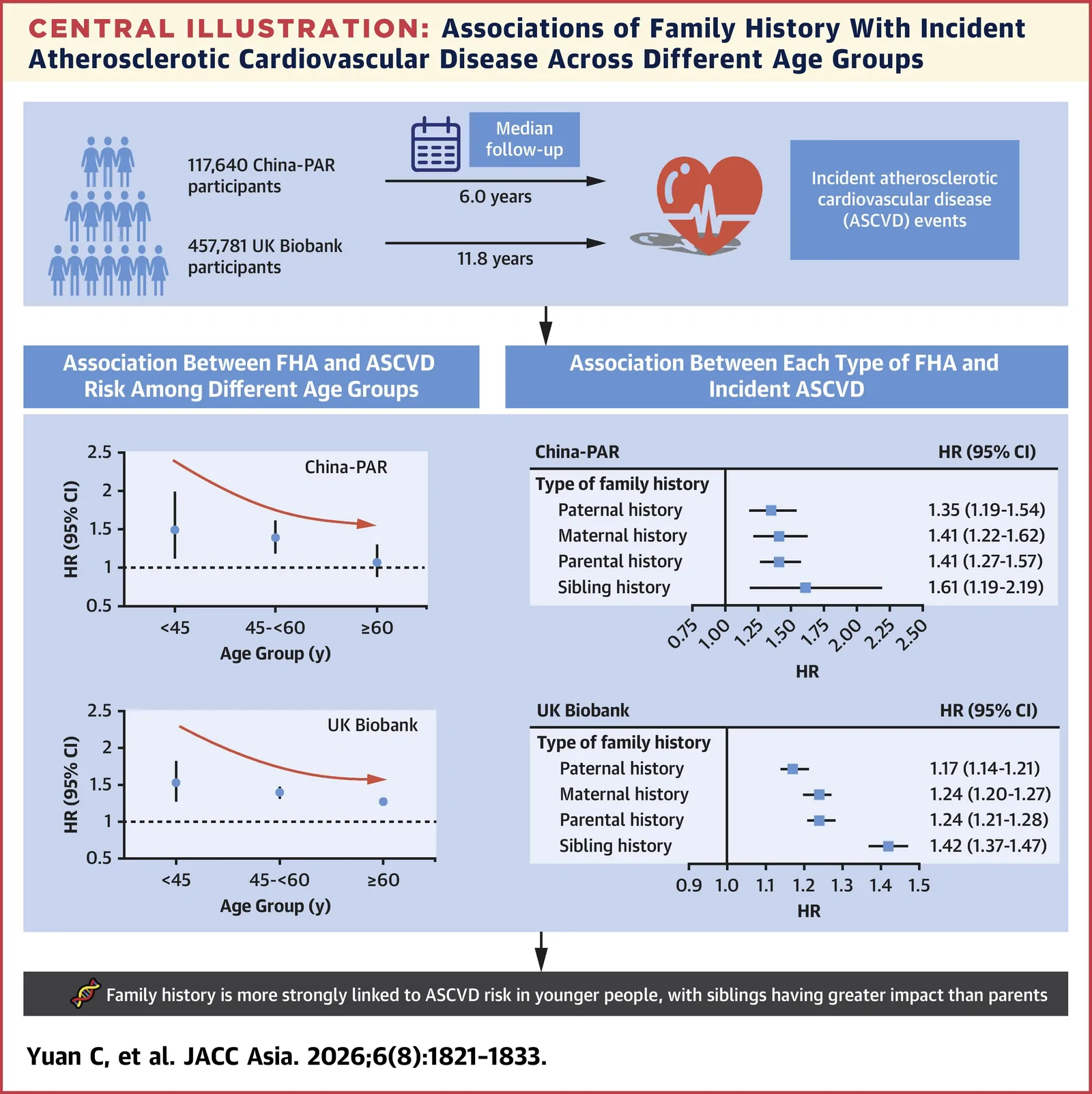
Associations of Family History With Incident Atherosclerotic Cardiovascular Disease Across Different Age Groups In 2 prospective cohort studies of 117,640 Chinese adults and 457,781 UK adults, the association between family history and atherosclerotic cardiovascular disease (ASCVD) risk is more pronounced in younger individuals, with sibling history having a greater impact than parental history. China-PAR = Prediction for Atherosclerotic Cardiovascular Disease Risk in China; FHA = family history of atherosclerotic cardiovascular disease.

### Comparison With Other Studies and Implications

The observed attenuation of the adverse impact of family history on ASCVD risk with increasing age is biologically plausible and supported by 2 potential explanations.^11^, ^12^, ^13^, ^14^, ^15^, ^16^^,^^36^ First, survivors with familial risk factors in the older age group may differ through selection. Those who are susceptible to family history are more likely to have had events earlier in life, and survival to older age with family history may be an indicator that they are less susceptible to such risk factors.^37^ Second, family history may serve as a warning factor, and that knowledge of a family history at a young age may be associated with a healthier lifestyle that gradually offsets the genetic risk as people grow older.^7^^,^^38^, ^39^, ^40^ Despite some skepticism, a study from Finland speculated that self-reported questionnaires of older adults may be less reliable than the younger adults due to the longer duration of first-degree relatives’ events, possibly leading to some degree of misclassification. Such misclassification may result in an underestimation of the true risk associated with a positive parental history, which could partially explain the differences in ASCVD risk associated with a positive family history between age groups.^12^ However, a study using a Danish national register, allowing the avoidance of different misclassifications of exposure and outcome, also found that the adverse effect of family history decreases with age.^13^ It is concluded that the effect of recall bias is not the main reason for the age difference in ASCVD risk. Overall, young individuals with a family history of ASCVD should be prioritized as key targets for cardiovascular risk reduction interventions. The study emphasizes the importance of enhanced health education and behavioral interventions in clinical practice for first-degree relatives of ASCVD patients, particularly younger individuals, as a potentially impactful policy option with significant public health implications. Future studies with larger sample sizes are warranted to further investigate the relationship between family history and ASCVD risk in even younger populations.

This finding is further supported by the negative coefficient of the multiplicative interaction term between FHA and age included in the China-PAR risk prediction model, which was previously published for primary prevention of ASCVD in the Chinese.^27^ Some other risk prediction tools, including Framingham, PCE (pooled cohort equations), and QRISK3 risk scores, also emphasize family history as a risk-enhancing factor,^10^^,^^41^^,^^42^ whereas it should be given different levels of importance across various age groups as indicated by this study, which could lead to improvements in risk prediction tools.

In addition, supported by the previous literature,^26^^,^^43^ our study found that sibling history was more strongly associated with the development and severity of ASCVD than parental history, filling a gap in developing countries' literature and overcoming prior research limitations through a prospective cohort design and comprehensive control of confounders. Family history is a combination of genetic factors and shared environmental factors, and previous studies have found that the influence of shared environmental factors on cardiovascular risk factors is stronger in siblings than in parent–offspring.^44^^,^^45^ One study from the China Kadoorie Biobank cohort also found that after adjusting for potential confounders (such as smoking and drinking status, education, etc), the numerical differences between sibling and parental medical history were reduced, suggesting that shared environmental factors may play an important role in the observed association between sibling history and outcomes, which highlights the influence of environmental exposures and lifestyle factors that siblings are more likely to share.^14^ However, the ability to disentangle genetic effects from shared environmental influences remains limited, as neither UK Biobank nor the China-PAR cohorts collected data on whether siblings lived together during the follow-up period. This lack of information restricts our ability to characterize the extent of environmental sharing and may introduce nondifferential misclassification, potentially biasing our findings toward the null. Therefore, although our results highlight the importance of sibling history in ASCVD risk stratification, they should be interpreted as indicative of overall familial risk, rather than reflecting purely genetic susceptibility. Future cohort studies with detailed household-level data may help to more precisely parse the relative contributions of heritable and environmental factors.

Taken together, these findings suggest that greater emphasis on sibling history could enhance cardiovascular risk prediction, particularly in younger individuals or those with multiple affected family members. At the same time, preventive strategies based on family history should consider both inherited predisposition and modifiable environmental exposures, ensuring a more balanced and actionable clinical interpretation.

### Strengths and Limitations of this Study

Major strengths of this study are the large sample size from 2 well-established nationwide cohorts in China and the United Kingdom—the findings are generally consistent between the 2 cohorts. The large sample size also allowed us to perform the joint and stratified analyses with sufficient statistical power. The prospective design and high rate of follow-up minimized the potential for loss to follow-up, enabling the capture of ASCVD morbidity events. We also conducted a series of sensitivity analyses to show the robustness of the findings. Reverse causality bias is unlikely in our study since we excluded individuals with existing ASCVD at baseline and included only the first episode of ASCVD after baseline measurement.

There are also some limitations that need to be mentioned. First, family history data were obtained through self-reported questionnaires, which may introduce some inaccuracies and misclassification bias, although previous studies have reported that self-reported history is reliable for cardiovascular disease data.^46^, ^47^, ^48^ However, such bias tends to favor the null hypothesis, potentially underestimating the effect size. Therefore, the estimates in this study are conservative, and the true impact of family history may be greater. Second, we did not obtain information about the age of onset of ASCVD in first-degree relatives of the participants. Therefore, we were unable to explore the association between early-onset ASCVD in first-degree relatives and incident ASCVD in the participants. Finally, although we controlled for key personal characteristics and metabolic factors, residual confounding was still possible, and causal inference cannot be made because of the nature of observational studies.

## Conclusions and Policy Implications

Based on 2 large nationwide cohorts in China and United Kingdom, we found that family history could provide significant value for individual ASCVD risk estimate, particularly among younger people. Notably, the impact of sibling history was more significant than that of parental history by comparing the results of different types of family history. Our findings suggest that early screening and targeted intervention among first-degree relatives of individuals with ASCVD, with a particular focus on younger individuals and those with sibling history, are of significant importance for the effective prevention of ASCVD in the United Kingdom and China.

### Data Sharing Statement

Data from China-PAR are available upon request, and data from UK Biobank are available on application at www.ukbiobank.ac.uk/register-apply.

## Funding Support and Author Disclosures

This work was supported by the National Science and Technology Major Program for Noncommunicable Chronic Diseases (grant numbers 2023ZD0503500 and 2025ZD0546500), the National Natural Science Foundation of China (grant numbers 82030102 and 82322059, T2541011), the Chinese Academy of Medical Sciences Innovation Fund for Medical Sciences (grant number 2021-I2M-1-010), National High Level Hospital Clinical Research Funding (grant numbers 2025-GSP-GG-32 and 2025-GSP-GG-36). The funders had no role in the study design or implementation; data collection, management, analysis, or interpretation; manuscript preparation, review, or approval; or the decision to submit the manuscript for publication. The authors have reported that they have no relationships relevant to the contents of this paper to disclose.
