# Supplementary material for: Family History and ASCVD Risk Among Different Age Groups: Cohort Study in China and the United Kingdom
Source: JACC Asia. 2026 Feb 20;6(8):1821–33. doi: 10.1016/j.jacasi.2025.10.031 (PMC13491090; doi:10.1016/j.jacasi.2025.10.031)
Supplement: Supplementary file 1 [file mmc1.docx]

**SUPPLEMENTAL APPENDIX**

**Supplemental Methods**

**Assessment of covariates**

In China-PAR, self-reported education level, occupation prestige, and household monthly per capita income were applied to assess SES according to previous studies. In UK Biobank, participants reported their education qualifications, employment status and total household income before tax. Each factor was categorized into low, medium, and high levels with consideration of practical interpretation and sample size within levels. We then respectively assigned 1, 2, and 3 points for the low, medium, and high levels of three SES factors, and participants’ points were summed up to generate SES score ranging from 3 to 9. Participants with a score of 3 to 5, 6, and 7 to 9 were categorized as low SES, medium SES, and high SES, respectively.

In China-PAR, the education level was divided into primary school or below, junior high school, and senior high school or above. The classification of occupational prestige is based on the Chinese occupational prestige score, determined by factors such as education level, income, authority, work unit model, and occupation subject to discrimination. Participants were classified as unemployed, low occupational prestige (prestige score <50, including workers, farmers, freelancers, other business and service personnel, and retirees), and higher (prestige score ≥50, including administrators, officials, professionals, and entrepreneurs). Since participants were recruited over a period of about 10 years, inflation might have contributed to incomparability of the sub-cohorts. Therefore, we calculated the income levels of sub-cohorts corrected by their corresponding income levels for 1998 through consumer price index. Monthly per capita income was quantified at three levels: <300 RMB/month, 300-800 RMB/month, and ≥800 RMB/month.

Participants in UK Biobank generally had higher levels of education than those in the Chinese. To be consistent with the distribution of education level in the Chinese, educational qualifications were classified into three groups according to previous studies: less than high school (O levels, GCSEs, or equivalent; CSEs or equivalent; None of the above), high school graduates (A levels, AS levels, or equivalent; NVQ, HND, HNC, or equivalent; other professional qualifications), and some college or above (college or university degree). Participants reported as prefer not to answer were excluded from our analyses as missing values. Based on the current employment status reported by the participants, we regrouped participants into three groups: low (including those in retired, looking after home and/or family, unable to work because of sickness or disability, and unemployed), medium (including those in doing unpaid or voluntary work, or being full or part time students) and high (including those in paid employment or self-employed). Annual total household income before tax was quantified at three levels: <£18,000 ($25,000; €21,000)-£30,999, £31,000-£100,000, and >£100,000. All questionnaires could be obtained through the UK Biobank website (https://biobank.ndph.ox.ac.uk/showcase/index.cgi).

**Supplemental Table 1.** Risk of incident ASCVD, stroke and CHD associated with family history of ASCVD according to age groups in the China-PAR and UK Biobank.

| **Group** | **Age group (yr)** | **China-PAR (n=117 640)** | | |  | **UK Biobank (n=457 781)** | | |
| --- | --- | --- | --- | --- | --- | --- | --- | --- |
|  |  | **Events/Total** | **Model 1** | **Model 2** |  | **Events/Total** | **Model 1** | **Model 2** |
| **ASCVD** |  |  |  |  |  |  |  |  |
|  | <45 | 545/40 976 | 1.91 (1.45 to 2.51) | 1.48 (1.11 to 1.96) |  | 658/44 829 | 1.82 (1.56 to 2.13) | 1.47 (1.23 to 1.76) |
|  | 45-<60 | 2030/50 245 | 1.57 (1.36 to 1.81) | 1.45 (1.25 to 1.68) |  | 7930/211 718 | 1.53 (1.46 to 1.60) | 1.43 (1.36 to 1.51) |
|  | ≥60 | 2106/26 419 | 1.15 (0.96 to 1.37) | 1.10 (0.92 to 1.31) |  | 18 325/201 234 | 1.33 (1.29 to 1.37) | 1.29 (1.25 to 1.34) |
| **Stroke** |  |  |  |  |  |  |  |  |
|  | <45 | 409/40 976 | 1.82 (1.32 to 2.53) | 1.45 (1.23 to 1.70) |  | 221/44 829 | 1.41 (1.08 to 1.95) | 1.29 (1.06 to 1.75) |
|  | 45-<60 | 1602/50 245 | 1.55 (1.32 to 1.82) | 1.36 (1.17 to 1.61) |  | 2640/211 718 | 1.36 (1.25 to 1.47) | 1.26 (1.16 to 1.37) |
|  | ≥60 | 1650/26 419 | 1.24 (1.03 to 1.51) | 1.20 (0.98 to 1.45) |  | 6829/201 234 | 1.17 (1.12 to 1.23) | 1.15 (1.09 to 1.22) |
| **CHD** |  |  |  |  |  |  |  |  |
|  | <45 | 144/40 976 | 2.24 (1.51 to 3.33) | 1.75 (1.17 to 2.63) |  | 461/44 829 | 2.18 (1.91 to 2.49) | 1.80 (1.55 to 2.10) |
|  | 45-<60 | 471/50 245 | 1.62 (1.27 to 2.07) | 1.45 (1.13 to 1.86) |  | 5884/211 718 | 1.60 (1.54 to 1.66) | 1.49 (1.43 to 1.55) |
|  | ≥60 | 481/26 419 | 0.84 (0.59 to 1.19) | 0.77 (0.54 to 1.10) |  | 13 677/201 234 | 1.34 (1.30 to 1.37) | 1.29 (1.25 to 1.33) |
| Stratified Cox proportional hazard model in China-PAR and Cox proportional hazard model in UK Biobank. Model 1: including sex. Model 2: adjusted for sex, current smoker, alcohol drinker, ideal physical activity, waist circumference, fasting glucose level (China-PAR) or hemoglobin A1c (UK Biobank), total cholesterol, high-density lipoprotein cholesterol, and systolic blood pressure.  ASCVD = atherosclerotic cardiovascular disease; CHD = coronary heart disease; China-PAR = Prediction for Atherosclerotic Cardiovascular Disease Risk in China; yr = year. | | | | | | | | |

**Supplemental Table 2.** Sensitivity analysis for association of ASCVD, stroke and CHD associated with family history of ASCVD among different age groups in the China-PAR and UK Biobank.

| **Analyses** | **Age group (yr)** | **China-PAR** | | |  | **UK Biobank** | | |
| --- | --- | --- | --- | --- | --- | --- | --- | --- |
|  |  | **ASCVD** | **Stroke** | **CHD** |  | **ASCVD** | **Stroke** | **CHD** |
| **Excluding incident ASCVD occurring within first year of follow-up** | | | | | | | |  |
|  | Total | 1.40 (1.26 to 1.56) | 1.42 (1.26 to 1.60) | 1.29 (1.08 to 1.55) |  | 1.33 (1.30 to 1.37) | 1.17 (1.11 to 1.23) | 1.37 (1.33 to 1.40) |
|  | <45 | 1.53 (1.15 to 2.04) | 1.43 (1.02 to 2.01) | 1.82 (1.22 to 2.74) |  | 1.47 (1.22 to 1.77) | 1.31 (1.07 to 1.79) | 1.80 (1.55 to 2.11) |
|  | 45-<60 | 1.43 (1.23 to 1.66) | 1.41 (1.19 to 1.67) | 1.46 (1.13 to 1.88) |  | 1.43 (1.36 to 1.51) | 1.29 (1.17 to 1.41) | 1.49 (1.43 to 1.56) |
|  | ≥60 | 1.09 (0.91 to 1.31) | 1.19 (0.97 to 1.45) | 0.75 (0.52 to 1.08) |  | 1.29 (1.25 to 1.34) | 1.14 (1.08 to 1.21) | 1.29 (1.25 to 1.33) |
| **Using Fine-Gray models instead, accounting for the competing risk of death** | | | | | | | |  |
|  | Total | 1.41 (1.27 to 1.57) | 1.44 (1.28 to 1.62) | 1.30 (1.09 to 1.55) |  | 1.35 (1.31 to 1.38) | 1.18 (1.13 to 1.24) | 1.37 (1.34 to 1.41) |
|  | <45 | 1.48 (1.11 to 1.98) | 1.44 (1.22 to 1.70) | 1.75 (1.15 to 2.66) |  | 1.44 (1.20 to 1.73) | 1.29 (1.05 to 1.75) | 1.80 (1.55 to 2.10) |
|  | 45-<60 | 1.46 (1.25 to 1.69) | 1.37 (1.16 to 1.62) | 1.46 (1.14 to 1.88) |  | 1.38 (1.31 to 1.45) | 1.26 (1.16 to 1.37) | 1.49 (1.43 to 1.56) |
|  | ≥60 | 1.11 (0.93 to 1.33) | 1.21 (1.00 to 1.48) | 0.78 (0.55 to 1.12) |  | 1.32 (1.27 to 1.36) | 1.16 (1.09 to 1.22) | 1.30 (1.26 to 1.34) |
| **Further adjusting for the socioeconomic status*** | | | | | | | |  |
|  | Total | 1.40 (1.26 to 1.55) | 1.44 (1.28 to 1.62) | 1.26 (1.06 to 1.51) |  | 1.34 (1.30 to 1.38) | 1.18 (1.12 to 1.24) | 1.37 (1.33 to 1.40) |
|  | <45 | 1.46 (1.10 to 1.94) | 1.45 (1.23 to 1.70) | 1.63 (1.08 to 2.46) |  | 1.43 (1.18 to 1.73) | 1.29 (1.05 to 1.75) | 1.77 (1.50 to 2.09) |
|  | 45-<60 | 1.45 (1.25 to 1.68) | 1.38 (1.01 to 1.91) | 1.42 (1.11 to 1.83) |  | 1.41 (1.33 to 1.49) | 1.26 (1.15 to 1.39) | 1.48 (1.41 to 1.55) |
|  | ≥60 | 1.11 (0.92 to 1.33) | 1.21 (0.99 to 1.47) | 0.79 (0.55 to 1.13) |  | 1.30 (1.26 to 1.35) | 1.16 (1.09 to 1.23) | 1.28 (1.24 to 1.33) |
| Cox proportional hazard models were stratified by cohort (just in China-PAR) and adjusted for age, sex, current smoker, alcohol drinker, ideal physical activity, waist circumference, fasting glucose level (China-PAR) or hemoglobin A1c (UK Biobank), total cholesterol, high-density lipoprotein cholesterol, and socioeconomic status systolic blood pressure. *Cox proportional hazard models were stratified by cohort (just in China-PAR) and adjusted for age, sex, current smoker, alcohol drinker, ideal physical activity, waist circumference, fasting glucose level (China-PAR) or hemoglobin A1c (UK Biobank), total cholesterol, high-density lipoprotein cholesterol, systolic blood pressure, and socioeconomic status. ASCVD = atherosclerotic cardiovascular disease; CHD = coronary heart disease; China-PAR = Prediction for Atherosclerotic Cardiovascular Disease Risk in China; yr = year. | | | | | | | | |

**Supplemental Figure 1.** Flowchart of sub-cohorts follow-up in the China-PAR project.

Abbreviations: China-PAR, Prediction for Atherosclerotic Cardiovascular Disease Risk in China; China MUCA, China Multi-Center Collaborative Study of Cardiovascular Epidemiology; InterASIA, International Collaborative Study of Cardiovascular Disease in Asia; CIMIC, Community Intervention of Metabolic Syndrome in China & Chinese Family Health Study.

**Supplemental Figure 2.** Directed Acyclic Graph representing the assumed relationships between the included variables.

No additional age adjustments were made in subgroup analyses stratified by age groups. Abbreviations: ASCVD, atherosclerotic cardiovascular disease; CHD, coronary heart disease.
